# Mistrust: Community engagement in global health research in coastal Kenya

**DOI:** 10.1177/03063127231162082

**Published:** 2023-04-01

**Authors:** Salla Sariola

**Affiliations:** University of Helsinki, Finland

**Keywords:** mistrust, global health research, clinical trials, community engagement, LGBTIQ+ activism

## Abstract

This article explores a case of mistrust in global health research and community engagement. It uses ethnographic material collected in 2014 and 2016 in Kenya, concerning community engagement by a HIV vaccine research group working with men who have sex with men and transgender women. In 2010, the research group was attacked by members of the wider community. Following the attack, the research group set up an engagement program to reduce mistrust and re-build relationships. Analysis focusing on mistrust shows the dynamics underlying the conflict: Norms around gender and sexuality, political support for LGBTIQ+ rights, and resources disparities were all at stake for those embroiled in the conflict, including researchers, study participants, religious leaders, and LGBTIQ+ activists in the region. Rather than a normative good with liberatory potential, community engagement in this paper is discussed as a relational tool with which mistrust was managed, highlighting the fragility of participation.

Community engagement as a form of participatory practice has proliferated in analyses of global health research, with trust as a crucial element in forging relationships between ‘science’ and ‘society’. Literature in science and technology studies (STS) has critically reflected on participatory practices in research and the democratic potential of participation. This article takes community engagement as the object of enquiry without a normative stance regarding its value. Instead, it asks questions that aim to open up engagement and trust to a critical enquiry: What does engagement *do*? Moreover, given the importance of engagement for building trust, how exactly does that work—what does it do in a context of mistrust? To understand trust and participatory practices, we should look at why community engagement is conducted in the first place.

I describe a case study in which a research group and its LGBTIQ+ study participants were attacked by members of the general community living in the study area. The article advances an argument about *mistrust* and the difficult social relationships that called for targeted community engagement. What happened here that required an engagement program to be set up? Mistrust is disruptive for global health research conduct; however, the reasons for conflict can go over and beyond research into broader societal and political dynamics. Building on feminist STS literature, I show how a combination of differing norms regarding gender and sexuality between researchers and local communities, poverty, and poor access to basic healthcare sparked a confrontation that then required engagement to re-build trust.

## The promise of engagement for global health

Community engagement, also at times described as ‘public engagement’, ‘patient and public involvement’, and ‘public participation’, is widely used in global health research, development programs, design, social policy, etc. Social scientific literature on participation has steadily increased: The 1990s and early 2000s saw a flurry of literature on participation that was critical of its implementation, especially within the development sector (Cooke & Kothari, 2002), and again in the late 2000s when UK government released a white paper on participation that mandated public participation (Felt & Fochler, 2010; Jasanoff, 2003; Stern & Green, 2008; Wynne, 2006). We can see a trajectory in which early science–society relations were characterized by a knowledge ‘deficit’ where knowledge was produced by elite experts, and publics were educated about it (Bauer et al., 2007). In the 2000s, the idea of the deficit was criticized by STS scholars who demonstrated that such an approach misses key issues and fails to recognize and incorporate multiple ways of knowing (Jasanoff, 2003; Wynne, 2006). Participatory approaches to knowledge production rapidly became key to good democratic governance of science, and several countries in Europe now mandate public participation in some contexts. Literature in STS exploring public participation is now so broad that this introduction cannot do justice to all of it (for a good review, see Kearnes & Chilvers, 2015).

An increase in engagement activities in global health research has stemmed from the struggles of international health research collaborations to overcome socio-economic differences and disparities underlying global medical research. Sites where research is conducted are often marked by poor infrastructure and limited primary health care (Geissler, 2013). International guidelines for biomedical research mandate community engagement as one of the key practices for making global health research more ethical (Lavery et al., 2010). Qualitative research from within public health has documented the institutionalization and mushrooming of global health engagement projects across the Global South (Reynolds & Sariola, 2018). Examples of engagement practices include: implementing consultations with community members (Marsh et al., 2008, 2010), enrolling peer educators and volunteers (Campbell et al., 2010; Sambakunsi et al., 2015), embarking on collaborations with activists and NGOs (Campbell et al., 2004; Versfeld et al., 2018), and using performances, theatre and other visual methods to communicate research findings (Aggett, 2018; Kombo et al., 2017; Lim et al., 2018). In practice, community engagement has also meant, for example, hearing social movements in health-related knowledge production (Campbell et al., 2010), and involving community members in the management of global health research in Community Advisory Boards (Maung Lwin et al., 2014; Nyirenda et al., 2018; Versfeld et al., 2018).

Contrary to a linear temporal trajectory of models of participation evolving towards more democratic practices over time, the current range of activities attached to global health research programs reflects a diverse, coexistent ecology of different kinds of engagement activities, where some participatory practices remain researcher-led and could be labelled as tokenistic and merely ‘involving’ communities, while others are bottom-up. There are examples of community-based research and patient activism in low-income countries, in which communities have halted exploitative drug research (Singh & Mills, 2005; Ukpong & Peterson, 2009), or where communities have driven their own health research agendas (Mosavel et al., 2005; Rhodes et al., 2012); these could be argued to be inclusive, and not controlled by researcher agendas. Analysing public participation around a pre-prophylaxis drug trial in Cambodia, Grant shows that in this low-income context, ‘epistemology and expertise were not the relevant or authoritative grounds for participation in scientific practices’ (Grant, 2016, p. 251). Traditional articulations of research ethics were insufficient to take local conditions into consideration and it was the activism by participating sex workers that pushed and renegotiated the relevant ethical principles so that bioethical practices were sensitive to their vulnerability, and human rights.

Contexts marked by under-developed health systems, precarious science governance structures, lack of protection of human rights, social stratification, and socio-economic differences between research elites and study participants create unique dynamics for how participation is implemented and what is at stake. I propose that there are relevant differences for participatory dynamics when situated in current international research structures and in contexts of broadening wealth gaps where global research programs are being conducted. The recent rise of community engagement in global health research programs makes it a re-vitalizing and fruitful yet unexplored object for STS analysis. A critical STS analysis of these global health community engagement programs remains limited, and this article brings the growing qualitative literature on community engagement together with STS analysis of mis/trust to an intersecting field of gender, sexuality, development, and overseas biomedical research.

## Conceptualizing trust and mistrust

The rhetorical purchase of engagement for global health research is considerable and begins to reveal how trust plays an important role in how engagement is seen as a solution to equalize global health discrepancies. A search of the global health literature with trust as a search-term showed that most papers on global health community engagement use trust as a key rationale to justify and explain engagement. Engagement is claimed to foster trust by, for example: increasing the resilience of health systems during epidemics by enhancing trust between communities, international organizations and local health authorities (Barker et al., 2020), increasing the likelihood that policy recommendations reflect the realities and constraints of policy and practice (McFadden et al., 2016), alleviating concerns about adverse events and research participation (Pell et al., 2019), encouraging innovation and fostering resilience in people and health systems (Odugleh-Kolev & Parrish-Sprowl, 2018), dissolving long-standing mistrust and suspicion among marginalized communities (Scharff et al., 2010; Usman et al., 2019), strengthening ethical conduct and mitigating the risk of further exploitation (Molyneux et al., 2005), fostering dialogue and learning between researchers and study communities (Wilkins, 2018), and establishing relationships and commitments with formal and informal authorities in the community (Lavery et al., 2010). This list is suggestive, more than exhaustive, but demonstrates the pervasiveness of trust and its normative promise in global health research.

Scholarship in STS highlights tools, methods, and scientific logics beyond health outcomes. As a crucial concept for the construction of science, trust has been explored in connection with collaboration (Shrum et al., 2001, 2007), the logic of clinical trials and their methodological procedures (Bijker et al., 2016; Fisher, 2008), the regulation of research (Hedgecoe, 2012), standards for research conduct (Timmermans, 2015), as well as the importance of trust for scientific unity bridging empirical and theoretical insights within scientific collectives (Reyes-Galindo, 2014). Trust in scientific authority, or the lack thereof, has highlighted the precariousness of the relationships of publics and scientific institutions (Aggrey & Shrum, 2020; Gauchat, 2012).

In developing an anthropology of trust, Corsin Jimenez (2005, 2011) asks what work trust does in societies, and in relationships beyond individuals (human and non-human alike).

I am interested in how knowledge, responsibility and social relationality have been organized as epistemologically distanced objects in contemporary capitalist regimes of audit and trust-making: objects liable of separation (moral crises) and mutual convergence (political ethics). Trust ﬁgures thus in this context as an engine of epistemic distance-compression: where knowledge, responsibility and mutuality collapse into an identical social form. (2011, p. 178)

Corsin Jimenez points to the functionality of trust for various contemporary institutions in societies that are broken from past social structures, in introducing new systems and organizations by which new relations are drafted—in this case, community engagement. However, the literature on trust does not shed light on the absence of trust: doubt, incredulity, scepticism, disconnectedness, or detachment, or the reasons for mistrust. Thus, focus on trust, I argue, only gets us so far.

I take inspiration from Carey’s (2017) work, which leads me to look at *mistrust* in global health engagement—not because, as Carey points out, because relationships are inherently ones between strangers and therefore not to be trusted, but rather because trust and mistrust are constitutive of one another. While trust is described as a social form that enables collaboration between trial participants and scientists, and between scientists themselves, mistrust can reveal what is at stake in global health relationships and engagement for the different partners involved.

Based on their multidisciplinary research on water purity in Mexico City, Leighton and Roberts (2020) argue that publics’ mistrust towards service providers is not due to ignorance but follows experiences of failures of infrastructure, precarity and corruption. Mistrust is not merely focused on the possible bacterial count of water, but holds together seemingly disparate elements from the surrounding society and ‘the geopolitical, religious, historical and economic processes that encompass uncertainty and transition’ (Leighton & Roberts, 2020, p. 7). Benjamin (2011) coins the term *sociality of distrust* when analyzing how African-American families with sickle cell disease feel about stem cell transplantation. Rather than seeing trust as something that individuals have or not, Benjamin points out that a crucial aspect in understanding mistrust is considering the histories that organize lack of trust, and in this she points to the legacy of medical institutions in failing to provide for Black communities. With this focus, organizations, rather than individuals, are objects of analysis. The legacy of the Tuskegee Trial in the US continues to influence public perception of biomedical research as well as the racialized assumption that Black communities are hesitant to take part in research (Benjamin, 2014). Rather than taking a deterministic and generalizing view of African-American views of research, Benjamin points to mismatches in health priorities and how advanced research is misplaced from needs of the African-American communities who are struggling with basic health care access (Benjamin, 2011, 2014). Similarly, on mistrust regarding HPV vaccines in Barbados, Charles (2022) argues that attitudes should not be parsed in terms of hesitation or resistance, but instead should be understood as *suspicion*: an ‘affective relation that circulates in the various socioeconomic, political, cultural, and historical formations that contextualize the vaccine, growing assemblages of multinational pharmaceutical networks and the state, and longer transnational histories of slavery, capitalist extraction, and public health’ (Charles, 2022, p. 13).

These feminist STS works on mistrust share the analytical stance that mistrust is not directed at the qualities or risks of a scientific product, but that it is expansive and relational, gathering various institutions, practices and actors, and results from social and historical contingencies. They bring attention to how race and sexuality produce and reproduce scientific organizations and their logics, as well as how they are seen by the public.

I propose that mistrust is a fruitful analytical way of thinking about global health engagement because mistrust illustrates the work done to ‘engage communities’ in global health research. Focusing on mistrust pushes the analysis beyond the normative promises of engagement and instead makes differences in values, expectations and stakes explicit. Mistrust helps us understand why and how, in the present case, the conflict erupted and unfolded the way it did. I show how trust and mistrust operate as relations rather than a binary: I use mistrust as a way of understanding the complex histories in biomedical research, and engagement, over differences concerning sexuality, economic opportunities, and power.

## Methods

I was embedded in research networks working on global health engagement for four years and conducted six months of participant observation in and interviews with members of the international AIDS vaccine research group in coastal Kenya in 2014 and 2016. As part of an international chain of HIV vaccine study sites, the group works to develop novel interventions to prevent HIV and AIDS: new vaccines and pharmaceuticals, social tools for improving drug adherence and encourage early HIV testing, and training health care practitioners.

During my fieldwork, I participated in the daily activities of the three site offices and interviewed a total of 30 people, which includes a majority of the staff of the research group: researchers, engagement practitioners, and counsellors (who often have overlapping roles). I further interviewed 22 peer educators, research participants and community-based organization members who were predominantly men between the age of 18-40 who had sex with men, but also included a few trans or lesbian women. These discussions were supplemented with informal conversations with staff working in the broader research institution of which the group was a part. Interviews concerned: the activities of the research group more generally, the challenges and ethics of vaccine research in the homophobic climate (at the time, the neighboring country Uganda had just introduced penalizing legislation against LGBTIQ+ that raised a lot of public debate in Kenya as well), the dynamics of gender and sexuality in the context, and life stories and experiences as a LGBTIQ+ person living in the region. The interviews lasted 30-90 minutes and were held in English or Kiswahili, with English interpretation by a member of the local queer community, and were transcribed and back-translated by a professional translator for validation. They were either recorded or noted as soon after as possible in field diaries.

I observed community engagement events concerning training for health care staff and dissemination of research findings (60 participants), national HIV task force meetings (20 participants), discussions about sexuality and objectives of HIV research with religious leaders (20 participants), LGBTIQ+ community members’ meetings (15 people across 5 organizations—hereafter, activist groups), and several recruitment events for ‘key populations’ held by engagement staff and researchers (attended by about 150 young gay men and trans women in total). I took part in organizing seven focus group discussions that included the screening of a film that the research group had made about its work, followed by a discussion on its utility in the context of rising conservatism around gender and sexuality in the East African region in 2014 after the neighboring country Uganda tightened its legislation on homosexuality (Bompani & Valois, 2017; Vorhölter, 2017). Material was analyzed using thematic analysis to look for recurring themes describing trust, mistrust, conflict, community engagement, and expectations for research and engagement. This was followed up by reiterative rounds of analysis looking for further articulations of difference in views of gender norms and sexuality, homophobia, research benefits, and social inequality and exemplary vignettes that would demonstrate those. My final analysis and interpretation are irredeemably partial. On the one hand, structurally I had a lot in common with many of the expatriate researchers in terms of having privileged academic training from a European university, relative wealth, and being white, all of which forged a sense of familiarity. On the other hand, my position as a queer female that became explicit in some of the ice-breaking exercises of the community engagement events contributed to a sense of trust among the queer interviewees that was essential for unraveling their version of the story. Nevertheless, I was an outsider not based at the research unit for the long-term. That suggest what Abimbola (2019) calls ‘foreign gaze’ and raises questions, in words of Karkey and Green (2018), about who can speak for whom in global health research. The manuscript was read by several colleagues at the unit in question, but the mistakes and interpretations are solely mine.

Study procedures were approved by the ethical review board at University of Oxford (application no: 1033-13) and the national research ethics committee in Kenya, and interviewees provided written or verbal informed consent.

## Case and context

The HIV research group is situated within an international global health research institute that works on various global health conditions. The institute has an established community engagement program that has made efforts to incorporate views of wider publics into its research, to help make that research culturally relevant and locally meaningful. The research group’s focus is on men who have sex with men (MSM) and trans women. The HIV research group was established in 2005, and was one of the first to be working with MSM in Africa. For the first decades of the fight against HIV, the focus in Africa was on heterosexual couples and parent-to-child transmission, and homosexual transmission was not prioritized. Patton (1993) has argued that in the history of AIDS in Africa, those who could not be configured in the ‘family model’ were ‘left to die’ by the AIDS industry in what she calls state-sponsored genocide—for a long time, the research group in Kenya fought against the idea that transmission between men was not an issue in Africa. The group initially set out to study prevalence of HIV among female sex workers, but through the networks of female sex workers it quickly became apparent that there was an active MSM community on the coast whose health care needs were not addressed by the general HIV prevention and care programs.

The group now has three research offices, one of which is in a buzzing trading town at the junction of several trucking roads, with busy markets, nightclubs, and performing artists of many kinds, where sex and sex work happens between local male and female sex workers and East African men, and European men and women, and where swinging couples from all over the world meet. While heterosexual commercial encounters are out in the open, there are no gay bars in the town and same-sex cruising remains hidden.

While HIV prevalence in the heterosexual population is decreasing in Kenya—at the time of writing at about 4.9% (National Aids Control Council, 2018)—the epidemic among MSM is considerably higher at 35% (Baral et al., 2007; Sanders et al., 2013; Smith et al., 2009). HIV research is challenged by the criminalization and stigma of same-sex relations in sub-Saharan Africa; MSM and trans women experience social stigmatization, mental health problems, sexual and physical violence, and poverty (Dijkstra et al., 2015; Secor et al., 2015).

In the research group, the senior staff included both Europeans and Kenyans with degrees in medical, natural and social sciences. In the organizational structure, next were engagement practitioners trained as social workers, and nurses from various Kenyan backgrounds. The staff came from diverse religious backgrounds, both Muslim and Christian, the predominant religions on the coast. The study participants and peer educators were men and trans women from Kenya or the neighboring countries Tanzania, Uganda, Somalia and Sudan from poor economic backgrounds who self-identified as *MSM*, *shoga, kuchu, queer*, and *gay*.

The second dynamic that characterizes this research unit is its encounters with and experiences of violence. In 2010, the office was attacked by an angry mob that gathered outside. Armed with stones, it threatened to set the clinic on fire and lynch those inside. It was a furious crowd of local residents who did not regularly visit the research unit, ignited by a Christian Clerk, a Muslim Imam, and regional politicians. The crowd was propelled by rumors according to which a gay wedding was going to take place in the area and that study participants, who were known to be homosexuals, could be found at the office. As the crowd raged at the gates of the office, the staff and some of the men inside escaped through the back windows, and the local police saved others from serious beatings by arresting them—they survived but with deep psychological trauma. A silence settled in the area, with staff and research participants distressed, and questioning the purpose of their efforts.

Later, one of the researchers explained that as part of the pre-prophylaxis trial that they were conducting, the research group provided study participants with breakfast—the absorption of the pharmacological compound required it and it was simultaneously a gesture towards benefits to participants. In the absence of safe public venues or sexual health services for MSM and trans women, the research office had become a safe space for those who took part in research, while it remained inaccessible to other members of the local community. For these others, it was explained to me, this looked like the research group was endorsing homosexuality or even paid young men to become homosexuals, a frightening prospect for many living in the area.

The attack made the researchers aware that they needed to work on their relations with this ‘outside’ community differently. Up to this point, the research group had involved high-risk group members and their advocacy organizations in a community advisory board but following the event, the research group felt the need to build relationships with community leadership more broadly, and to gain their trust. Local and national support was needed to be able to continue the work, in fact, the community acceptance was vital to be able to continue at all. They established a broader engagement program that took into consideration the need to connect with the wider, general community living in the area.

## Communities and engagement practices

In an interview that took place four years after the attack, a former trial participant explained that many participants felt an elevated sense of stigma and blame for ‘causing’ the incident. The interviewee, who had been a study participant and peer educator at the time, described it as follows:

Actually, after the incident, staff felt that, you know, that MSM can really land them into problems. Their clinic can be burned. They can be beaten by the general community. They thought that we were responsible for all this mess. Some staff didn’t want MSM to be treated in the research. They didn’t want MSM to be recruited as staff. I remember my supervisor back then said these people can really land you run into problems. So on the one hand, they really depend on MSMs for their research, and at the same time …

The attack led to concerns about continuing the work. One of the researchers explained: ‘We closed the office to think through our operations. It was a difficult time. To continue or not? While the work is super important, we also didn’t want to put our staff and participants at risk’. In the end, research activities were re-launched, now with social workers in place to run an engagement program. An engagement program helped to forge and mediate relationships with communities within which the research group worked. Community was defined by one of the engagement practitioners as:

Fluid, and there is no standard definition. For me, a community is actually people, any kind of people. You have a gay community, we have a general community, we have religious leaders’ community. … So it really depends on who your target is. These are just people that you are interested in targeting for specific endeavor.

Another engagement practitioner described engagement as a way of giving attention to various groups that have a potential stake in the research but also to those that have the power to interfere with the research, of which there were many. Engagement activities tap into existing networks but also build new ones, depending on project needs (see also Montgomery & Pool, 2017).

As a result of ‘sustained listening’, a voluntary counselling and testing (VCT) center was opened adjacent to the clinical trials unit; this was for the general community to access STI tests and to receive treatment. While in practice anyone could be a member of the public who would benefit from these activities, the work of engagement practitioners focused specifically on two groups: health care practitioners and religious leaders.

Targeted as potential allies by the research group were health care professionals, nurses and doctors in the regional hospitals and health care centers, and the local HIV Task Force. Health care practitioners were addressed via training events that the HIV research group had developed, and research dissemination events. In these struggling health systems, care for MSM and trans women was poorly delivered, due to a lack of awareness of the health needs of this community, as well as common outright stigma. The online training program that the research group had developed, endorsed by the National AIDS and STI Control Program under the Ministry of Health and available to anyone across Africa working in similar circumstances, included modules on: the history of MSM and HIV in Sub-Saharan Africa; homophobia, stigma and its effects; sexual identity and coming out; anal sex and common sexual practices; HIV and STIs, condoms and lubricants; mental health, depression, anxiety and substance use; and risk reduction counseling (Dijkstra et al., 2015). By the end of 2014, nearly 1000 health care practitioners had completed the training and the feedback from that was very positive, despite the very contentious issues and practices that were included (van der Elst et al., 2013; van der Elst, Kombo et al., 2014).

As part of its engagement activities, the research group held various sessions to report on its research activities to senior staff of local hospitals. The meetings served to both convey to health care practitioners information about most recent research evidence, as well as to inform the researchers about health care practitioners’ views regarding HIV and the work of the group. The meetings were also an opportunity to address practitioners’ views that might prevent them from providing the best possible care. The engagement events, then, served to build relationships and trust that went in both directions, towards researchers and towards the health care professionals, linking the international research group with local national health services. While learning about the special needs of MSMs and trans women, health care professionals may have absorbed a de-stigmatizing attitude, as well as notions about the right to non-normative sexuality. While at times these messages were contested and homophobic and hateful comments were aired, the developing relationships were primarily characterized by and built on professional collegiality.

Engagement of religious leaders, on the other hand, was described as essential for alleviating *mistrust*. Following a systematic engagement program with those who had generated hatred and sparked the mob, several central figures of the attack became more positive towards the study group. One of the engagement practitioners described the importance of engagement as ensuring that community understandings were in line with what the group did, rather than what Marsh et al. (2011) term ‘half-knowing’, and that the research group had some control over the discourse about them in the public domain. An engagement practitioner explained:

Before, when the community tried to attack us, we didn’t have any engagements. People used to believe in rumors and meetings that were held outside. So one of the key strategies was to identify the religious leaders, police, the key people, and to bring them on board, tell them what we do, and to say, this is what we do in our meetings. And then they give us feedback from the community. Since then, I feel we have made a huge step. These are the people who wanted to ‘man’ us (beat, teach a lesson).

Engagement activities included workshops with those individuals that had been part of the attack and also others who had similar roles in the society. At these workshops, religious leaders reflected on the implications of their interpretations of Christian and Islamic dogmas to the HIV prevention and treatment agenda. At one of the workshops I attended, they raised questions about connections between homosexuality and Satanism, if insatiable women were witches, and what caused homosexuality. As the engagement program ensued, reasons behind the attack became clearer. In the next sections, I will elaborate on the issues that underlined the conflict and contributed to the mistrust concerned.

## Homophobia, gender, sexuality, and ‘Africanness’

Underlying the mistrust were clashes in values regarding sexuality, concerning the sexual health of minorities vis-à-vis religious-cultural heteronormativity. I asked one of the engagement practitioners what the wider community members were concerned about.

It’s not easy to convince the community that we do not promote homosexuality. What we do, it is all ‘health-wise’. Most people think that we are giving them (LGBTIQ+ persons) services, meaning that we are emphasizing people to continue doing gay-ism and promote homosexuality. We have addressed that through community engagement, but we haven’t reached every point in the community. It’s not easy, it’s not easy at all. The group still has a bad name outside.

SS: And why is that?

It’s not easy to change someone’s mind and it takes time. People feel that if we know someone is gay, why are we giving them services? Why are we promoting homosexuals? These people are not required in the community. According to community perspective, it’s against the Bible and it’s against the Quran. God created man to marry women and not man to man to have sex. So according to them, it’s like Satanic behavior. The fact that this is a high-risk area, some of the parents are against what we are doing because they feel that our children, their sons, may also be involved in homosexuality. They fear that.

Several interviewees identified differing ideas about sexuality and normative sexual behavior between the researchers and the local communities. Expatriate researchers embraced a harm-reduction approach and supported a right to sexuality. What was referred to as local customs, most vocally by religious leaders, assumed a gender binary defined by sexuality in relation to an opposite sex and with regard to procreation and having children. The tolerant attitudes towards LGBTIQ+ that the research endorsed were seen to be in conflict and to undermine profound family and religious values of the local community. This is a deeply patriarchal society where men have power and prestige in the family and women take care of growing food, carrying water, and looking after children. It is customary in the Swahili, Masai and Muslim traditions that men have several wives, and law in Kenya sanctions polygamy. Homosexuality, in contrast, was described as abnormal, Satanic, un-Christian, un-Islamic, and against local customs and norms. During engagement meetings, there was a burgeoning understanding of the importance of the need to prevent HIV but homophobic and hostile comments were regularly aired nonetheless.

These values were not limited to the private domain but were supported by national anti-homosexual legislation and connected to broader postcolonial ideology. When, in 2015, U.S. President Barack Obama visited Kenya, he commented on the lack of rights of LGBTIQ+ people in Kenya and publicly requested that Kenya recognize gay rights—the penal code institutionalized during British colonial rule had made ‘non-natural carnal relations’ illegal. Obama’s talk was met with conservatism, hostility and national resentment. In response, Kenyan President Uhuru Kenyatta stated that Kenyans have other priorities, and that homosexuality is Western and un-African. It was a non-issue for Kenyans, Kenyatta declared. In May 2019, the Kenyan court decided to uphold the law that criminalizes homosexuality, referring to cultural and religious norms.

The argument that homosexuality is un-African taps into international postcolonial ideology. Anti-homosexuality is aggressively fueled by American ultra-conservative Christian evangelic movements strong in coastal areas. The fact that the research organization was working with MSM was evidence of ‘Western collusion’ to the protesters, who saw the researchers as introducing homosexuality to the area. For example, Gichuru et al. (2018) quote one of the Muslim leaders, who had been a ringleader in the clinic attack saying: ‘The lack of local involvement in these initiatives shows that organizations in Africa are funded by Western countries to spread homosexuality.’

Within this climate, those who failed to conform to a strict gender binary, especially in public, risk their health, safety, and life. I asked one of the staff members at the research unit, a young gay man working as a counsellor, about coming out in this context. He saw value in the liberated, out-and-proud approach to homosexuality that international LGBTIQ+ discourse promotes, and which the European researchers embraced, but deemed it a risky strategy in Kenya.

Being gay is criminalized in Kenya, so people should be responsible. … Disclosing here is something which is risky because of the people who are dominant here. Here I feel I might be at risk, I have experienced several attacks, I even have some marks behind here so I know what it’s like. I was born and raised here so I know how coastal people can react—I can easily be killed. So disclosing here in a gathering, it’s something that I am not sure that I can do. Not for now at least. I disclose today and after three hours they will come and burn me.

Wanting to understand the dynamics more deeply, I asked what he thought people were afraid of or angry about.

I don’t know if it’s more of the culture here or the religion, I don’t really know. But surprisingly, everyone is aware that it [same-sex relations] is happening. I think the issue is that if you stand and disclose in front of people, it will be like you’re provoking them. It will be like you are boasting your sexuality. And that is something that they don’t like. Passing by a group of people in the villages where Muslims are staying, even if you are cross-dressed, it’s not a big deal. But again, disclosing in a gathering it will be like you are proud of yourself and they might get angry.

In such a context, the idea of coming out as emancipatory, and the commonly used practice in HIV prevention programs and LGBTIQ+ human rights campaigns of ‘disclosing’ personal stories for various audiences, usually in exchange of a small reward for contributing to the event, is confronted with challenges. It was common to start meetings for MSM and other most-at-risk populations with a norm change exercise and declaration ‘My name is X and I am (sexual practice definition of one’s choice)’. This was often met with ice-breaking laughter, but also nervousness and tension. Nguyen (2005) has described how these portrayals of sexuality and identity in HIV organizations in Burkina Faso contributed to the development of therapeutic citizenship through shared struggle and recognition. While Nguyen points to the potential for the recognition of rights and new empowered subjectivities through such occasions, in this context of post-conflict, there was a high chance that such declarations were read differently. Coming out was deemed dangerous and getting paid for participating in engagement events where such declarations were made seen as corruptive of local values. Being seen to be ‘paying people to be gay’ (Biruk, 2020) puts the economic situation squarely in view.

## Material differences

The research headquarters was located in a small town on the coast—a 45-minute drive from the attacked office, and in a different township—idyllic, tropical landscape with lush greenery, exquisite white beaches, and turquoise ocean dotted with boats and yachts. Staff lived in the town center, or further afield in houses or apartments with better infrastructure, while the research participating men and trans women lived in rented apartments that were often just one room if they lived alone, or rural family compounds further away if they lived with their families—often without running water, sewage, or electricity. The research institute was protected by fences and barbed wire, its gates guarded by armed security staff and accessible only with staff cards or invitations. The geographical demarcation signals class differences between those involved in research; writing from Western Kenya, Geissler (2013) has termed these socio-economic differences the ‘unknown knowns’ whereby the differences between the staff in overseas biomedical research collaborations and the research participants are bracketed out from everyday operations, discourses and scientific reports, while at the same time they are the very *raison d’être* of global health research. It is for these poverty-related diseases that global health research is conducted. Geissler concludes that in the absence of an open debate about these dynamics, speculation of partially known disparities can translate into concealed misgivings and animosities, translated into rumours of the occult, or seek legal outlets (Geissler, 2013, p. 18). I argue that in addition to homophobia, the other key factor that contributed to why and how things unfolded concerns socio-economic differences and finances.

While researchers saw that they were endorsing the international LGBTIQ+ cause and alleviating social suffering of this very vulnerable group, this support was seen by many as ignoring the majority in the region, because everyone was poor. One of the engagement practitioners cited material differences as the reason why the wider communities had acted against the research group; she explained that people wanted to have their share of the wealth that the research center was seen to have. The research institution of which the HIV group was part was the biggest employer in the area and those associated with it were regularly approached with requests for personal loans to support children’s school fees, organizations, investments for small businesses, jobs, etc.

Engagement events provided formalized channels to translate financial benefits to individuals, which helped to alleviate the sense of mistrust. Community engagement forged an on-going interpersonal and informal relationship between individual religious leaders and the engagement practitioners: The religious leaders regularly came to spend time at the research office, having a bite to eat and a chatting with engagement practitioners, who also met the leaders in their places of worship. There was always food available, and participants got a small, non-coercive sum of money as compensation for attending (5-10$).

Benefits from participating were not restricted to immediate financial interests; relations with the research group could be leveraged for future opportunities and kudos. Those religious leaders who had the closest relationships with the unit regularly took part in engagement events aimed at other groups, such as meetings with the National HIV Task Force. For religious leaders, becoming a spokesperson for powerful HIV research institute gained them visibility and even international attention as exceptional African leaders. In one engagement meeting, Christian religious leaders described their commitment to the HIV cause via Christian scriptures that emphasized ‘bringing back lost sheep’, underscoring values of mercy and kindness to the ‘fallen’ group.

The metaphor continues to rely on the othering of queer people, and over several meetings that I observed, it became evident that the endorsement of some of the community leaders was ambivalent at best. One preacher, for instance, who in one meeting embraced homosexual men as equals, said in another that homosexuality was a sin and that he did not approve of it but encouraged people to come out of it. The feedback from many LGBTIQ+ community activists as well as some researchers deemed the support as unpredictable.

Engagement with gay men consisted of activities that aimed at sustaining relationships with MSM and trans women who could potentially become participants in vaccine trials and other studies in the future. A small group of peer educators spent time at the study office on a daily basis. Their experiences and identities as young queer men made them valuable to the project because of their knowledge of networks of other men at high risk of HIV and who could be recruited for the research. They kept in touch with others via social media apps, went out to distribute condoms in the evenings to nearby bars, sought new men into the circles of the research group and gave them advice about STI and HIV protection. For many young men and trans women, their interest in research was to gain access to free health checks that they were able to have at the study center. The (potential) study participants were economically vulnerable; in the absence of stable economic opportunities, a large portion of those whom I interviewed had exchanged money for sex. While individually, the incentive for engagement events may not seem much when translated to donor dollars (roughly 5$), it was on par with or more than what some sex workers received in exchange for sex. Travel reimbursements were a valuable additional source of income that the study participants, peer educators, community advisory board members, community mobilizers, religious leaders, and other engaged individuals with modest incomes had come to rely on (for a similar argument about per diems, see Conteh & Kingori, 2010). Compensation was strategically gauged by the researchers in consultation with various local community groups—while a valuable additional income in contrast of many riskier income sources, it was also considered important that the compensation for engagement events would not be coercive to participation in experimental research.

Many of the research volunteers were connected with LGBTIQ+ activist groups. At the time of my research, there were eight community-based organizations (CBOs) on the coast. LGBTIQ+ groups on the coast invariably state as their main focus the social and developmental needs of the LGBTIQ+ people. They advocated condom use, peer education, and safety of LGBTIQ+. The main agenda of all CBOs was to create equal rights for those who had non-normative sexual relations, the right to sexuality and free sexual and relational expression. The coastal activist organizations are part of Galck, the umbrella organization of LGBTIQ+ rights in Kenya based in the capital Nairobi, which ‘promotes recognition, acceptance and the interests and rights of LGBTIQ+ organizations and their members’ Some of the CBOs’ activities were financed by the research group, and they collaborated regularly in dissemination and training events. After the attack, support by the medical researchers for LGBTIQ+ lobby groups was intentionally made inexplicit, partly in fear of further threats to their operations and of putting staff and participants at risk. In public, agendas of activist organizations were kept separate from those of research.

Some activists commented on the separation of agendas in interviews and expressed that they wanted to be more included in research and wished the research organization could support them politically in public. One of them exclaimed:

You come, you’ve been told something, you sign a consent form, you are given an incentive (compensation for attendance), and then you participate and that is it. But now, as a community-based organization, we realize actually that most of our members are jobless. And they are not engaged in research to be employed. How come they are not given opportunities in terms of employment? I feel that if these people are not fully engaged, they are being used as guinea pigs. But if they were fully engaged on all kinds of aspects from up to the bottom then we are set to go. I feel we earn it. It is not you who owns it, but we earn it.

The interviewee felt that because researchers were not addressing needs beyond health, participants were reduced to exploited trial subjects. This claim, unique in its strong terms, demonstrates both the financial differences between research subjects and researchers but also that the mistrust can go in many directions. The comment linked absence of public support in all aspects of lives of participants with exploitative human subject research as an exchange that goes well beyond what bioethical guidelines stipulate. It articulates tensions between financial (under)privileges, and some of the expectation for the research group. Some activists hoped that they could have the support of the powerful group of researchers for their international contacts, funds, and employment opportunities, and the absence of that undermined trust in the group and ideas of ethical research participation. Since the interview was held, activists and researchers have authored work together regarding research ethics, consolidating their discussions regarding good research conduct that highlight the vulnerability of (potential) research participants, financially and otherwise (van der Elst et al., 2017; van der Elst, Gichuru et al., 2014).

Researchers described that the work that they had to do to navigate (mis)trust and the expectations of finances, political support and international networks, landing on them from all directions, turned research into development. The expectations went beyond scientific questions at hand, into social improvements beyond what biomedical research traditionally delivers. While the researchers personally wanted to do everything they could, and stood behind decriminalizing homosexuality, they felt they had to limit their support to working on health research because, institutionally, they were restricted by the apprehensions of the wider organization towards openly emphasizing LGBTIQ+ research in the context of a broad health research agenda. Given that the group was part of a parastatal organization, the researchers were concerned about acting against their institutional mandate and ultimately the Kenyan state. It was feared that joining a legal cause to overthrow the Kenyan penal code could be seen unconstitutional—an irredeemable position for a parastatal organization, and one that could again escalate the situation with the wider community. Here, then, the engagement emerges as a continuous balancing act between the right to health and right to sexuality, and maintaining relationships with all relevant parties in the region in the context of financial socio-economic discrepancies. Engagement provided a space to air and negotiate these diverse needs and differences that was crucial for overcoming mistrust.

## Conclusion

Most global health research continues without controversy. Attacks against medical research institutions in low-income settings in Africa are not, however, exceptional events, but have resulted from various religious, socially, politically and culturally contingent dynamics. Indeed, conflicts fueled by mistrust between researchers associated with power-holding groups and resistance by members of wider populations have occurred in the history of medical institutions throughout colonial and postcolonial eras (Geissler, 2005; Graboyes, 2015; Tappan, 2014; Wilkinson & Fairhead, 2017). Graboyes and Carr (2016) examine institutional memory concerning scientific failure in East Africa and argue that because of the projectification of medical research there are gaps in institutional recall about these conflicts—amnesia fueled by a ‘heroism narrative’ of scientists working ‘against odds’ and a need to prove the ‘novelty’ of each project.

Contrary to the research on community pushback reported from elsewhere, the confrontation here was not to question top-down knowledge and structures of expertise (Hastings, 2016; Wynne, 2001), or sparked by concerns about structural violence and coercion that would put participants at risk in international research (Fairhead et al., 2006a; Grant, 2016; Nyirenda et al., 2020). Such controversies have ultimately had the outcome of expanding the types of knowledge recognised as legitimate and relevant, liberalizing research. Looking at conflict with a feminist science and technology studies framing offers explanations for mistrust, and what was at stake for the different participants embroiled in the controversy; medical research becomes an arena for broader political questions and social concerns, and conflicts are produced and reproduced by existing social values, inequalities, and structures that go over and beyond the individuals involved (Benjamin, 2011, 2014; Leighton & Roberts, 2020). What I have shown is a story of contingent, differing norms in gender and sexuality, and socio-economic interests and opportunities that fueled mistrust.

Mistrust provided a justification for violence and continued discrimination of LGBTIQ+, where the first crucial point of tension concerned more progressive and internationally informed definitions of LGBTIQ+ rights vis-à-vis patriarchal and heteronormative, religiously reasoned norms regarding gender and sexuality entrenched in postcolonial political ideology. The second point of contention I have highlighted concerns the differences in socio-economic opportunities between the researchers and the people living in and around the research institute—be they (potential) research participants or members of the wider public.

In this mistrustful context, it was possible to rebuild some relationships after the conflict, via converging interests. Differences were negotiated through various practices of engagement: Engagement was everyday labor that paved a way from conflict to working relations and offered a way to know each other. In Corsin Jimenez’s (2005, p. 193) terms, trust-building was mobilized ‘against dark forces of secrecy, uncertainty and risk’. Engagement practices rendered hitherto unknown relationships knowable, enabling operations of research, and created conditions for aligning differing interests. The content or subject matter of the engagement activities was important but more important were the relationships that were formed with publics.

In that sense, mis/trust was not a permanent state but a relation where there could be more or less trust and that could be worked on. Engagement was nebulous enough for various groups to inscribe into it a range of interests, and socially open-ended enough for engagement practices to sustain them to alleviate mistrust. Despite the differing needs, aims and objectives, engagement activities created spaces for the different members to gain kudos and benefits of various kinds, ranging from financial benefits to elevated social positions and leverage for other social and political agendas. Thus, engagement served as the tightrope on which different interests were negotiated and aligned to manage mistrust. Engagement served as the ‘distance-compression’ (Corsin Jimenez, 2011, p. 178) between research elites, expatriates, local, often rural, and/or impoverished communities, etc. whose values and backgrounds were otherwise almost irredeemably different. In this example, public participation eventually resulted in research that was more inclusive of broader publics *but not via intentions or means that were democratizing or emancipatory.* Simply focusing on the outcomes of engagement would mask these tensions and thus remain precarious.

In this case, the story of engagement is complicated and contested, but ultimately with positive outcomes. The success masks a final point worth spending a moment on: What does community engagement do in global health programs more broadly, when enabling trust between various interest groups? The gaps in public and primary health care systems that international collaborations and bilateral funders are filling in many low-income countries have been left behind by structural adjustments rolling back the state implemented across Asia and Africa (Biehl & Petryna, 2013; Pfeiffer, 2003). The benefits of international collaborations are temporally fraught, as they depend on project funding. The work of engagement to create and maintain trust, then, is framed by environments in which short-term research cannot replace the universal health care coverage that would be needed to ensure continuous health care access. While engagement and global health research can be harnessed to improving health systems, the rifts created by broader economic structures will continue to influence health research activities and create circumstances that give rise to mistrust. Until political economies within which health care and research are situated are addressed, unequal power dynamics will inevitably be read in and on global health projects, to which engagement cannot be the solution. To ‘do engagement better’ does not call for more engagement. The extent to which involving local communities can democratize research and/or radically alter the direction of global research and health care access is limited by design; to do that, a more critical look at the broader political economies of research and the global relations within that, is required. We need to look at those structures that generate mistrust to begin with.
